# Performance of Hamiltonian Monte Carlo and No-U-Turn Sampler for estimating genetic parameters and breeding values

**DOI:** 10.1186/s12711-019-0515-1

**Published:** 2019-12-10

**Authors:** Motohide Nishio, Aisaku Arakawa

**Affiliations:** Institute of Livestock and Grassland Science, NARO, 2 Ikenodai Tsukuba, Ibaraki, 3050901 Japan

## Abstract

**Background:**

Hamiltonian Monte Carlo is one of the algorithms of the Markov chain Monte Carlo method that uses Hamiltonian dynamics to propose samples that follow a target distribution. The method can avoid the random walk behavior to achieve a more effective and consistent exploration of the probability space and sensitivity to correlated parameters, which are shortcomings that plague many Markov chain Monte Carlo methods. However, the performance of Hamiltonian Monte Carlo is highly sensitive to two hyperparameters. The No-U-Turn Sampler, an extension of Hamiltonian Monte Carlo, was recently introduced to automate the tuning of these hyperparameters. Thus, this study compared the performances of Gibbs sampling, Hamiltonian Monte Carlo, and the No-U-Turn Sampler for estimating genetic parameters and breeding values as well as sampling qualities in both simulated and real pig data. For all datasets, we used a pedigree-based univariate linear mixed model.

**Results:**

For all datasets, the No-U-Turn Sampler and Gibbs sampling performed comparably regarding the estimation of heritabilities and accuracies of breeding values. Compared with Gibbs sampling, the estimates of effective sample sizes for simulated and pig data with the No-U-Turn Sampler were 3.2 to 22.6 and 3.5 to 5.9 times larger, respectively. Autocorrelations decreased more quickly with the No-U-Turn Sampler than with Gibbs sampling. When true heritability was low in the simulated data, the skewness of the marginal posterior distributions with the No-U-Turn Sampler was smaller than that with Gibbs sampling. The performance of Hamiltonian Monte Carlo for sampling quality was inferior to that of No-U-Turn Sampler in the simulated data. Moreover, Hamiltonian Monte Carlo could not estimate genetic parameters because of difficulties with the hyperparameter settings with pig data.

**Conclusions:**

The No-U-Turn Sampler is a promising sampling method for animal breeding because of its good sampling qualities: large effective sample sizes, low autocorrelations, and low skewness of marginal posterior distributions, particularly when heritability is low. Meanwhile, Hamiltonian Monte Carlo failed to converge with a simple univariate model for pig data. Thus, it might be difficult to use Hamiltonian Monte Carlo for usual complex models in animal breeding.

## Background

In the 1980s, Gianola and Foulley [1] and Gianola and Fernando [2] introduced Bayesian inference methods to animal breeding. Although Bayesian methods were theoretically powerful, they encountered difficulties in mathematical computation. Bayesian methods usually led to formulas in which multiple integrals had to be solved in order to obtain the marginal posterior distributions that are used for a complete Bayesian inference. These computation problems were solved by applying Markov chain Monte Carlo (MCMC) methods, which could simulate direct draws from target posterior distributions without analytically solving multiple integrals. Accordingly, MCMC methods were introduced to quantitative genetics in the early 1990s [3, 4] and enabled the widespread use of Bayesian methods in animal breeding.

One of the most general MCMC methods is the Metropolis–Hastings (MH) algorithm. MH simulates candidate samples from arbitrary proposal distributions that are approximations of the target distribution, and then corrects for the bias by stochastically accepting or rejecting the proposal to satisfy the detailed balance. In MCMC, choosing an appropriate proposal distribution is a critical issue to accelerate convergence with the smallest number of samples. However, the choice is problem-dependent. Gibbs sampling (GS) [5, 6] is another MCMC method and is a special case of MH. GS repeatedly samples from the conditional distribution of one variable of the target distribution when all the other variables are given [7]. GS is frequently used in practice because a proposal distribution does not need to be designed and the procedure is simple to program. However, GS cannot be applied to complex models in which growth curve parameters and environmental variance are under genetic control [8–10], because conditional distributions cannot be derived in such models.

In this context, Hamiltonian Monte Carlo (HMC) is an increasingly popular alternative MCMC method. HMC adopts Hamiltonian dynamics in physics to propose future states in the Markov chain. Hamiltonian dynamics in HMC allows the Markov chain to simulate arbitrarily long trajectories in parameter space around the target distribution. Thus, HMC can theoretically generate samples from a wide range of parameter space with a high level of acceptance probability. However, the success of HMC is due to geometric numerical integration of Hamiltonian dynamics, which is markedly affected by the two hyperparameters. A poor choice of parameters decreases the efficiency of HMC dramatically [11].

To mitigate the challenges of tuning the abovementioned hyperparameters, Hoffman and Gelman developed the No-U-Turn Sampler (NUTS) [11]. NUTS uses a recursive algorithm to automatically tune the HMC algorithm without requiring user intervention or costly tuning runs. NUTS algorithms have recently been packaged into Stan (a probabilistic programming language) [12, 13] and the BGLIMM procedure in SAS. Stan is used in social science [14], pharmaceutical statistics [15], and ecology among others [16].

GS is widely used to estimate genetic parameters and breeding values. Although HMC and NUTS are also prospective methods for animal breeding, these methods have not yet been applied in this field. Therefore, this study compared the performance of GS, HMC, and NUTS for estimating genetic parameters and breeding values with both simulated and real pig data.

## Methods

### Hamiltonian Monte Carlo method

HMC is a variation of the Metropolis algorithm that uses Hamiltonian dynamics to create proposals. In a physical system, the Hamiltonian ($H$) is defined as follows:

$$H\left( {{\varvec{\uptheta}},{\mathbf{p}}} \right) = U\left( {\varvec{\uptheta}} \right) + K\left( {\mathbf{p}} \right),$$

where $U\left( {\varvec{\uptheta}} \right)$ and $K\left( {\mathbf{p}} \right)$ are the potential and kinetic energies, respectively. The property of the dynamics is that it keeps the $H$ invariant.

When estimating a random variable ${\varvec{\uptheta}}$ with probability density function *f*$\left( {\varvec{\uptheta}} \right)$ in the HMC method, we define an auxiliary momentum variable ${\mathbf{p}}$ that follows a normal distribution: $f\left( {\mathbf{p}} \right) \sim N\left( {0,{\mathbf{M}}} \right)$, where ${\mathbf{M}}$ is interpreted as a covariance matrix in statistics. The joint density function of $f\left( {{\varvec{\uptheta}},{\mathbf{p}}} \right)$ has the following form:

$$f\left( {{\varvec{\uptheta}},{\mathbf{p}}} \right) = \exp \left\{ {\log f\left( {\varvec{\uptheta}} \right) + \log f\left( {\mathbf{p}} \right)} \right\} \propto \exp \left( {\log f\left( {\varvec{\uptheta}} \right) - \frac{1}{2}{\mathbf{p^{\prime}M}}^{ - 1} {\mathbf{p}}} \right).$$

In HMC, $U\left( {\varvec{\uptheta}} \right)$ and $K\left( {\mathbf{p}} \right)$ are defined as $U\left( {\varvec{\uptheta}} \right) = - \log f\left( {\varvec{\uptheta}} \right)$ and $K\left( {\mathbf{p}} \right) = {\mathbf{p^{\prime}M}}^{ - 1} {\mathbf{p}}/2$, respectively. Thus, the joint density function of $f\left( {{\varvec{\uptheta}},{\mathbf{p}}} \right)$ can be rewritten as follows:

$$f\left( {{\varvec{\uptheta}},{\mathbf{p}}} \right) = \exp \left\{ { - U\left( {\varvec{\uptheta}} \right) - K\left( {\mathbf{p}} \right)} \right\} = { \exp }\left\{ { - H\left( {{\varvec{\uptheta}},{\mathbf{p}}} \right)} \right\}.$$

HMC generates samples $\left( {{\varvec{\uptheta}},{\mathbf{p}}} \right)$ from this joint distribution, and then we can obtain the samples from the target distribution by picking up only ${\varvec{\uptheta}}$. According to Hamiltonian dynamics, the samples are moved while maintaining the total energy, which is described by the following two differential equations, a so-called Hamilton’s equation:

$$\frac{{d{\varvec{\uptheta}}}}{dt} = \frac{\partial H}{{\partial {\mathbf{p}}}},$$

$$\frac{{d{\mathbf{p}}}}{dt} = \frac{\partial H}{{\partial {\varvec{\uptheta}}}},$$

where $t$ is the fictitious time. However, there is no analytical solution for Hamilton’s equation; therefore, Hamiltonian dynamics is usually approximated in a discrete time setting to enable computer implementation. The discretization integration for HMC generally uses the leapfrog method, which provides a good approximation for Hamiltonian dynamics. The leapfrog method can preserve the two important properties for Hamiltonian dynamics, “reversibility” and “volume preservation,” which rely on the use of MCMC updates [17]. The leapfrog integration proceeds as follows:

$${\mathbf{p}}\left( {\tau + \frac{1}{2}\varepsilon } \right) = {\mathbf{p}}\left( \tau \right) - \frac{1}{2}\varepsilon \frac{{\partial f\left( {\varvec{\uptheta}} \right)}}{{\partial {\varvec{\uptheta}}}}\left( {{\varvec{\uptheta}}\left( \tau \right)} \right),$$

$${\varvec{\uptheta}}\left( {\tau + \varepsilon } \right) = {\varvec{\uptheta}}\left( \tau \right) + \varepsilon {\mathbf{p}}\left( {\tau + \frac{1}{2}\varepsilon } \right),$$

$${\mathbf{p}}\left( {\tau + \varepsilon } \right) = {\mathbf{p}}\left( {\tau + \frac{1}{2}\varepsilon } \right) - \frac{1}{2}\varepsilon \frac{{\partial f\left( {\varvec{\uptheta}} \right)}}{{\partial {\varvec{\uptheta}}}}\left( {{\varvec{\uptheta}}\left( {\tau + \varepsilon } \right)} \right),$$

where $\varepsilon$ is the integration step size and $\tau$ is the time ($1 \le \tau \le L$). These integration steps are replicated until $\tau$ reaches $L$, which is the number of integration steps in the leapfrog method. For one integration step, we start with a half-step for ${\mathbf{p}}$, then perform a full step for ${\varvec{\uptheta}}$, using the new values for ${\mathbf{p}}$. Starting from the state $\left( {{\varvec{\uptheta}},{\mathbf{p}}} \right)$, the proposal state $\left( {{\varvec{\uptheta}}^{ *} ,{\mathbf{p}}^{*} } \right)$ is reached via $L$ steps of step size $\varepsilon$ in the leapfrog method. In the leapfrog method, $H$ is not exactly conserved because of the integration error caused by the time discretization. Therefore, a Metropolis correction step is necessary to ensure correct sampling. In this correction step, the proposed state $\left( {{\varvec{\uptheta}}^{ *} ,{\mathbf{p}}^{*} } \right)$ is accepted as the next state of the Markov chain with the following probability:

$$\alpha = { \hbox{min} }\left\{ {1,{ \exp }\left( {H\left( {{\varvec{\uptheta}},{\mathbf{p}}} \right) - H\left( {{\varvec{\uptheta}}^{ *} ,{\mathbf{p}}^{*} } \right)} \right)} \right\} ,$$

which corresponds to the Metropolis–Hasting acceptance probability. If the integration errors in $H$ remain small during the integration, HMC will achieve a high level of acceptance probability.

The difficult point of HMC is that the sampling efficiency relies heavily on tunings for the two user-defined hyper parameters: $\varepsilon$ and $L$ [11]. On the one hand, a large value of $\varepsilon$ leads to a low acceptance rate due to an increase of the integration error by the leapfrog integration. In contrast, if $\varepsilon$ is too small, a long computation time will be needed to obtain the adequate trajectory length. On the other hand, the number of steps $L$ affects sampling efficiency; if $L$ is too small, samples generated by HMC show quite high autocorrelations between successive iterations. In contrast, a large $L$ leads to a large trajectory length, which may move the parameters back to their original states.

### No-U-Turn Sampler

NUTS automatically selects an appropriate value for $L$ in each iteration in order to maximize the distance at each leapfrog step and avoid random-walk behavior. Let $Q$ be half the squared distance between the current position ${\varvec{\uptheta}}^{*}$ and the initial position ${\varvec{\uptheta}}$ at each leapfrog step. The aim is to run leapfrog steps until ${\varvec{\uptheta}}^{*}$ starts to move back towards ${\varvec{\uptheta}}$, which is accomplished by the following algorithm, in which leapfrog steps are run until the derivative of $Q$ with respect to time becomes less than 0:

$$\frac{\partial Q}{\partial \tau } = \frac{\partial }{\partial \tau }\frac{{\left( {{\varvec{\uptheta}}^{ *} - {\varvec{\uptheta}}} \right)^{'} \left( {{\varvec{\uptheta}}^{ *} - {\varvec{\uptheta}}} \right)}}{2} = \left( {{\varvec{\uptheta}}^{ *} - {\varvec{\uptheta}}} \right)^{'} {\mathbf{p}} < 0.$$

However, this algorithm does not guarantee “reversibility” or convergence to the correct distribution. NUTS overcomes this problem by applying a doubling method for slice sampling [18].

Slice sampling is an MCMC method for sampling from a probability distribution. To obtain samples of $\theta$ from the target distribution $f\left( \theta \right)$, we introduce an auxiliary variable $u$ and a joint distribution $f\left( {u,\theta } \right)$. This joint distribution is defined as follows:

$$f\left( {u,\theta } \right) = \left\{ {\begin{array}{*{20}l} {{\raise0.7ex\hbox{${ 1}$} \!\mathord{\left/ {\vphantom {{ 1} z}}\right.\kern-0pt} \!\lower0.7ex\hbox{$z$}}} & \quad {{\text{if}} \, 0 \le u \le \pi \left( \theta \right)} \\ 0 & \quad {\text{otherwise}} \\ \end{array} } \right.,$$

where $z = \smallint \pi \left( \theta \right)d\theta$ and $\pi \left( \theta \right)$ is a kernel of $f\left( \theta \right)$. The marginal distribution of $f\left( {u,\theta } \right)$ is as follows:

$$\smallint f\left( {u,\theta } \right)du = \mathop \smallint \limits_{0}^{\pi \left( \theta \right)} \frac{1}{Z}du = \frac{\pi \left( \theta \right)}{Z} = f\left( \theta \right).$$

Therefore, we can sample $\theta$ from the target distribution by sampling from $f\left( {u,\theta } \right)$, and then ignoring $u$. In slice sampling, these procedures are accomplished by alternately sampling $u$ and $\theta$. In the first step, we fix $\theta$ and sample $u$ uniformly to satisfy $u \le \pi \left( \theta \right)$:

$$p(u| \theta ) \sim Uniform\left( {0,\pi \left( \theta \right)} \right).$$

Then, we fix $u$ and sample $\theta$ uniformly from the horizontal sliced region $S$ defined by:

$$S = \left\{ {\theta : u \le \pi \left( \theta \right)} \right\}.$$

In the slice sampling algorithm, the challenge is to find the bounds of $S$. Therefore, Neal [18] proposed a doubling method in which the size of an initial segment containing the current value of $\theta$ is randomly chosen and the segment is expanded by doubling its size until the endpoints are outside $S$. The expanding directions are randomly chosen from forward or backward. A subset of candidate $\theta$ is obtained from the segment generated by the doubling process.

NUTS begins by introducing $u$ with the following uniform distribution:

$$p(u| {\varvec{\uptheta}}) \sim Uniform\left( {0,\exp \left( {\log f\left( {\varvec{\uptheta}} \right) - \frac{1}{2}{\mathbf{p^{\prime}M}}^{ - 1} {\mathbf{p}}} \right)} \right).$$

NUTS generates a finite set of all $\left( {{\varvec{\uptheta}},{\mathbf{p}}} \right)$ by repeatedly doubling its size. Doubling proceeds by randomly taking forward and backward leapfrog steps to satisfy time reversibility. The doubling process is stopped to satisfy the following:

$$\left( {{\varvec{\uptheta}}^{ + } - {\varvec{\uptheta}}^{ - } } \right)^{ '} {\mathbf{p}}^{ - } < 0 \; or \; \left( {{\varvec{\uptheta}}^{ - } - {\varvec{\uptheta}}^{ + } } \right)^{ '} {\mathbf{p}}^{ + } < 0,$$

where ${\varvec{\uptheta}}^{ + }$, ${\mathbf{p}}^{ + }$ and ${\varvec{\uptheta}}^{ - }$, ${\mathbf{p}}^{ - }$ are the leftmost and rightmost ${\varvec{\uptheta}}$, ${\mathbf{p}}$ of all $\left( {{\varvec{\uptheta}},{\mathbf{p}}} \right)$ generated by the doubling process, respectively. Here, let $C$ be a subset of candidate $\left( {{\varvec{\uptheta}},{\mathbf{p}}} \right)$ states. In NUTS, $C$ is selected from the $\left( {{\varvec{\uptheta}},{\mathbf{p}}} \right)$ generated by the doubling process to satisfy the following:

$$u \le \exp \left( {\log f\left( {\varvec{\uptheta}} \right) - \frac{1}{2}{\mathbf{p^{\prime}M}}^{ - 1} {\mathbf{p}}} \right).$$

The next values of $\left( {{\varvec{\uptheta}}^{ *} ,{\mathbf{p}}^{*} } \right)$ are sampled uniformly from $C$. To further improve this algorithm, Hoffman and Gelman [11] used the following sophisticated transition kernel in each step of doubling:

$$T\left( {{\varvec{\uptheta}}^{ *} ,{\mathbf{p}}^{*} |{\varvec{\uptheta}},{\mathbf{p}},C} \right) = \left\{ {\begin{array}{*{20}l} {\frac{{I\left[ {{\varvec{\uptheta}}^{ *} ,{\mathbf{p}}^{*} \in C^{new} } \right]}}{{\left| {C^{new} } \right|}}} & {when \quad \left| {C^{new} } \right| > \left| {C^{old} } \right|} \\ {\frac{{\left| {C^{new} } \right|}}{{\left| {C^{old} } \right|}}\frac{{I\left[ {{\varvec{\uptheta}}^{ *} ,{\mathbf{p}}^{*} \in C^{new} } \right]}}{{\left| {C^{new} } \right|}} + \left( {1 - \frac{{\left| {C^{new} } \right|}}{{\left| {C^{old} } \right|}}} \right)I\left[ {\left( {{\varvec{\uptheta}}^{ *} ,{\mathbf{p}}^{*} } \right) = \left( {{\varvec{\uptheta}},{\mathbf{p}}} \right)} \right]} & {when \quad \left| {C^{new} } \right| \le \left| {C^{old} } \right|} \\ \end{array} } \right.,$$

where $I\left[ \cdot \right]$ is 1 if the expression in brackets is true and 0 if it is false, $C^{new}$ is the subset of $\left( {{\varvec{\uptheta}},{\mathbf{p}}} \right)$ added by the last step of doubling, and $C^{old}$ is the disjoint subset of $C$ such that $C = C^{new} \cup C^{old}$ and $\left( {{\varvec{\uptheta}},{\mathbf{p}}} \right) \in C^{old}$. This transition kernel $T$ proposes a move from $C^{old}$ to a random state in $C^{new}$, and accepts the move with probability ${{\left| {C^{new} } \right|} \mathord{\left/ {\vphantom {{\left| {C^{new} } \right|} {\left| {C^{old} } \right|}}} \right. \kern-0pt} {\left| {C^{old} } \right|}}$. In leapfrog steps, $T$ permits memory-efficient implementation and produces larger jumps on average than simple uniform sampling.

The efficient implementation of NUTS relies on the acceptance probability. When the acceptance probability is too high, the step size is small, resulting in many leapfrog steps being needed to generate subset $C$. Hoffman and Gelman [11] reported that an acceptance probability of 0.6 was the optimal balance. NUTS can automatically choose a step size that achieves an acceptance probability around the desired level [19], which is one of the stochastic optimizations. The process of tuning $\varepsilon$ for the *j*th iteration of a Markov chain in NUTS is as follows:

$$\log \left( {\varepsilon_{j + 1} } \right) \leftarrow \mu - \frac{\sqrt j }{\gamma }\frac{1}{{j + j_{0} }}\mathop \sum \limits_{i = 1}^{j} \left( {\delta - \alpha_{j} } \right),$$

$$\log \left( {\overline{\varepsilon }_{j + 1} } \right) \leftarrow \eta_{j} { \log }\left( {\varepsilon_{j + 1} } \right) + \left( {1 - \eta_{j} } \right)\log \left( {\overline{\varepsilon }_{j} } \right),$$

$$\varepsilon_{j + 1} \leftarrow \overline{\varepsilon }_{j + 1} ,$$

where $\alpha_{j}$ is an actual acceptance probability for the *j*th iteration, $\delta$ is a desired average acceptance probability, $\mu$ is a freely chosen point that the iterated $\varepsilon_{j}$ shrink towards, $\gamma$ is a free parameter that controls the amount of shrinkage towards $\mu$, and $j_{0}$ is a free parameter that dampens early exploration. Hoffman and Gelman [11] introduced $\eta_{j} = j^{ - \kappa }$ and set $\kappa < 1$, which give a bigger weight to more recent iterates. They recommend setting $\mu = { \log }\left( {10\varepsilon_{1} } \right)$ and $\delta \approx 0.6$. This algorithm guarantees that $\alpha \to \delta$. In NUTS, $\varepsilon$ is tuned during the predetermined warm-up phase and is fixed thereafter. Because NUTS chooses $\left( {{\varvec{\uptheta}}^{ *} ,{\mathbf{p}}^{*} } \right)$ from multiple candidates, an alternative statistic to Metropolis acceptance probability must be defined. For each iteration, the acceptance probability is calculated as follows:

$$\alpha_{j} = \frac{1}{{\left| {B_{j} } \right|}}\mathop \sum \limits_{{{\varvec{\uptheta}},{\mathbf{p}} \in B_{j} }} min\left\{ {1,\frac{{p\left( {{\varvec{\uptheta}}^{j} ,{\mathbf{p}}^{j} } \right)}}{{p\left( {{\varvec{\uptheta}}^{j - 1} ,{\mathbf{p}}^{j,0} } \right)}}} \right\},$$

where ${\varvec{\uptheta}}^{j}$ and ${\mathbf{p}}^{j}$ are the candidates, ${\varvec{\uptheta}}^{j - 1}$ and ${\mathbf{p}}^{j,0}$ are initial values, and $B_{j}$ is the set of all states explored during the final doubling for the *j*th iteration of the Markov chain.

The sampling procedure by NUTS is summarized as follows:

1. Set the initial value of ${\varvec{\uptheta}}$, $\varepsilon$ and values of $\delta$, $\mu$, $\gamma$, $j_{0}$, $\kappa$.
2. Generate momentum ${\mathbf{p}}$ from the standard normal distribution ${\mathbf{p}} \sim N\left( {0,{\mathbf{I}}} \right)$.
3. Generate auxiliary variable $u$ from the uniform distribution $u \sim Uniform\left( {0,\exp \left( {\log f\left( {\varvec{\uptheta}} \right) - \frac{1}{2}{\mathbf{p^{\prime}M}}^{ - 1} {\mathbf{p}}} \right)} \right)$.
4. Generate $C$ by using the doubling method with transition kernel $T$.
5. Accept the proposal $\left( {{\varvec{\uptheta}}^{ *} ,{\mathbf{p}}^{*} } \right)$ with probability $\alpha_{j}$ at the *j*th iteration.
6. Update $\varepsilon_{j}$ by dual averaging.
7. Repeat steps (2) to (6). Note that step (6) is repeated only during the warm-up phase.

For a precise definition and a pseudocode of the NUTS algorithm, see Hoffman and Gelman [11].

### Statistical model

The following univariate linear mixed model was used:

$${\mathbf{y}} = {\mathbf{Xb}} + {\mathbf{Za}} + {\mathbf{e}},$$

where ${\mathbf{y}} = n \times 1$ is the observation vector ($n$: number of records), ${\mathbf{b}} = p \times 1$ is the vector of fixed effects ($p$: number of fixed effects), ${\mathbf{a}} = q \times 1$ is the vector of direct additive genetic effects ($q$: number of animals), and ${\mathbf{e}} = n \times 1$ is the vector of residuals; ${\mathbf{X}}$ and ${\mathbf{Z}}$ denote the incidence matrices relating the observations to the corresponding fixed and random effects. The likelihood of the model and prior distributions for ${\mathbf{a}}$ can be specified as ${\mathbf{y}}|{\mathbf{b}},{\mathbf{a}},\sigma_{e}^{2} \sim N\left( {{\mathbf{Xb}} + {\mathbf{Za}},{\mathbf{I}}\sigma_{e}^{2} } \right)$ and ${\mathbf{a}}|\sigma_{a}^{2} \sim N\left( {0,{\mathbf{A}}\sigma_{a}^{2} } \right)$, respectively, where ${\mathbf{A}}$ denotes the pedigree-based additive genetic relationship matrix, and $\sigma_{a}^{2}$ and $\sigma_{e}^{2}$ are the variances for ${\mathbf{a}}$ and ${\mathbf{e}}$, respectively. The prior distribution for ${\mathbf{b}}$ is assumed to be a uniform distribution.

Partial derivatives for log posterior $f\left( {\varvec{\uptheta}} \right)$ with respect to each parameter are required in leapfrog procedures in HMC or NUTS. Here, let ${\varvec{\uptheta}}$ be the vector of parameters ${\mathbf{b}}$, ${\mathbf{a}}$, $\sigma_{a}^{2}$, and $\sigma_{e}^{2}$. Partial derivatives of $f\left( {\varvec{\uptheta}} \right)$ with ${\varvec{\uptheta}}$ are expressed as follows:

$$\frac{d}{{d{\mathbf{b}}}}\log f\left( {\varvec{\uptheta}} \right) = \frac{1}{{\sigma_{e}^{2} }}{\mathbf{X}} '\left( {{\mathbf{y}} - {\mathbf{Xb}} - {\mathbf{Za}}} \right),$$

$$\frac{d}{{d{\mathbf{a}}}}\log f\left( {\varvec{\uptheta}} \right) = - \frac{1}{{\sigma_{a}^{2} }}{\mathbf{A}}^{ - 1} {\mathbf{a}} + \frac{1}{{\sigma_{e}^{2} }} {\mathbf{Z}} '\left( {{\mathbf{y}} - {\mathbf{Xb}} - {\mathbf{Za}}} \right),$$

$$\frac{d}{{d\sigma_{a}^{2} }}\log f\left( {\varvec{\uptheta}} \right) = - \frac{q}{{2\sigma_{a}^{2} }} + \frac{{{\mathbf{a^{\prime}A}}^{ - 1} {\mathbf{a}}}}{{2\sigma_{a}^{4} }},$$

$$\frac{d}{{d\sigma_{e}^{2} }}\log f\left( {\varvec{\uptheta}} \right) = - \frac{n}{{2\sigma_{e}^{2} }} + \frac{1}{{2\sigma_{e}^{4} }}\left( {{\mathbf{y}} - {\mathbf{Xb}} - {\mathbf{Za}}} \right)^{'} \left( {{\mathbf{y}} - {\mathbf{Xb}} - {\mathbf{Za}}} \right).$$

### Simulated data

The simulated data were generated by using QMSim [20]. Under an infinitesimal model, the base population comprised 20 males and 100 females generated from a historical population with linkage equilibrium. After the base population, the next five generations were generated to investigate the performance of HMC and NUTS. For these five generations, one male was randomly selected as a sire of the next generation and mated to 10 females to produce 10 males and 10 females. Total population sizes were set to 1000 with an equal number of each sex. The heritabilities of the simulated phenotypes were set to 0.1, 0.3 or 0.5, and phenotypic variance was assumed to be 1. For each condition, five replicates were simulated. In the statistical analysis, the fixed effect was sex effects.

### Pig data

Pig data were derived from 1521 purebred Duroc pigs at the National Livestock Breeding Center, Japan. Pigs in the first and second generations were regarded as the base population, and closed breeding was subsequently performed from the third to seventh generation. The pigs were selected based on average daily gain from 30 to 105 kg, backfat thickness (BF), loin eye area (LEA), and intramuscular fat content. BF and LEA were measured on the left side at 9 cm from the position of half the body length in pigs weighing 105 kg using ultrasound (B-mode) equipment (USL-11, Kaijo-denki Co., Ltd., Tokyo). This study focused on 991 records of BF and LEA. Descriptive statistics for the BF and LEA data are in Table 1. In the statistical analysis, the fixed effects were sex effects (three classes: boar, barrow, and gilt) and generation effects (seven classes).

**Table 1:** Descriptive statistics for growth traits in Duroc pigs

Trait	Mean	SD	Minimum	Maximum
BF (cm)	3.17	0.56	1.76	5.04
LEA (cm^2^)	34.53	3.49	25.97	51.03

*BF* back fat thickness, *LEA* loin eye area, *SD* standard deviation

### Heritability estimation and accuracy of estimated breeding values

The computing programs for GS and HMC were developed in R, and the programs for NUTS were developed in Stan. Developing a program for NUTS is challenging because of its very complex algorithm but it can be overcome by using Stan, which involves a simple programming language. Stan is an open-source software, with a publicly available manual online (https://mc-stan.org/users/documentation/). In the present study, we used RStan, which is the R interface for Stan. The pseudocode for NUTS is described in Additional file 1. Stan implements reverse-mode automatic differentiation to calculate the gradients of the model, which is required by the leapfrog steps of HMC and NUTS. Therefore, the user can implement NUTS by merely writing the analysis model. In this pseudocode, we convert the additive relationship matrix by Cholesky decomposition to identify a matrix to describe an animal model in Stan format. This improves the performance of MCMC. Thus, for a fair comparison, we also run the Cholesky decomposition of the additive relationship matrix for GS and HMC. In HMC, we assumed that ${\mathbf{M}} = {\mathbf{I}}$, where ${\mathbf{I}}$ is an identity matrix. In Stan, we can define the value of ${\mathbf{M}}$. When ${\mathbf{M}}^{ - 1}$ approximates the covariance matrix of the target distribution, the kinetic energy function, $\frac{1}{2}{\mathbf{p^{\prime}M}}^{ - 1} {\mathbf{p}}$, will reduce the negative impacts of strong correlations and bad scaling on the efficiency of HMC and NUTS. For the default setting of the Stan software, ${\mathbf{M}}$ is defined as a diagonal metric (i.e., a diagonal matrix with positive diagonal entries) and the values of ${\mathbf{M}}$ are estimated during the warm-up phase [13].

All records were used to estimate heritabilities and breeding values with both simulated and pig data. The posterior mean and Monte Carlo standard error (MCSE) were calculated to evaluate the estimated heritabilities. The MCSE describes the uncertainty about a statistic in the sample due to sampling error. For the accuracy of estimated breeding values, we calculated Pearson’s correlation between the true value and the estimated breeding value. The regression coefficient of the true value on the estimated breeding value of animals was calculated to assess unbiasedness. The true value was assumed as the true breeding value in the simulated data and the phenotype in the pig data.

In total, 10,000 iterations were simulated to obtain marginal posterior distributions for all methods. For GS and HMC, the first 1000 iterations were discarded as burn-in. The warm-up phase was 1000 iterations for NUTS. For HMC, the hyperparameters were set as follows: $\varepsilon = 0.01$ and $L = 100$ in the simulated data, and $\varepsilon = 0.001 \sim 10$ and $L = 3 \sim 200$ in the pig data. For HMC, the initial values of ${\varvec{\uptheta}}$ were sampled from the uniform distribution $Uniform\left( {0,{\mathbf{I}}} \right)$. For NUTS, we used default parameters in Stan.

### Performance of MCMC

We presented the traceplots and marginal posterior distributions of heritability estimates to evaluate the performance of MCMC samplings for all methods. The effective sample size (ESS) and autocorrelation between samples were calculated by using the R “coda” package [21]. For autocorrelation by using the function “acf” on R, the intervals between samples were set to 1, 5, 10, and 50 (lag1, lag5, lag10, and lag50, respectively).

## Results

### Simulated data

**Fig. 1:**
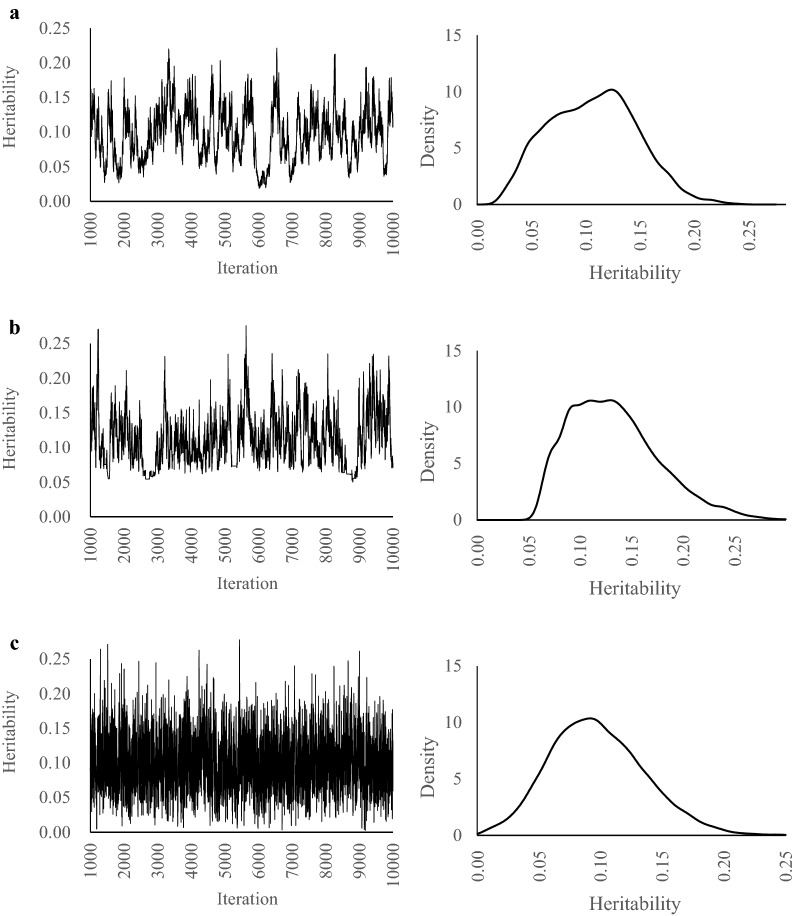
Trace plots and posterior density plots for heritability. **a** GS, **b** HMC, and **c** NUTS. True heritability = 0.1

**Fig. 2:**
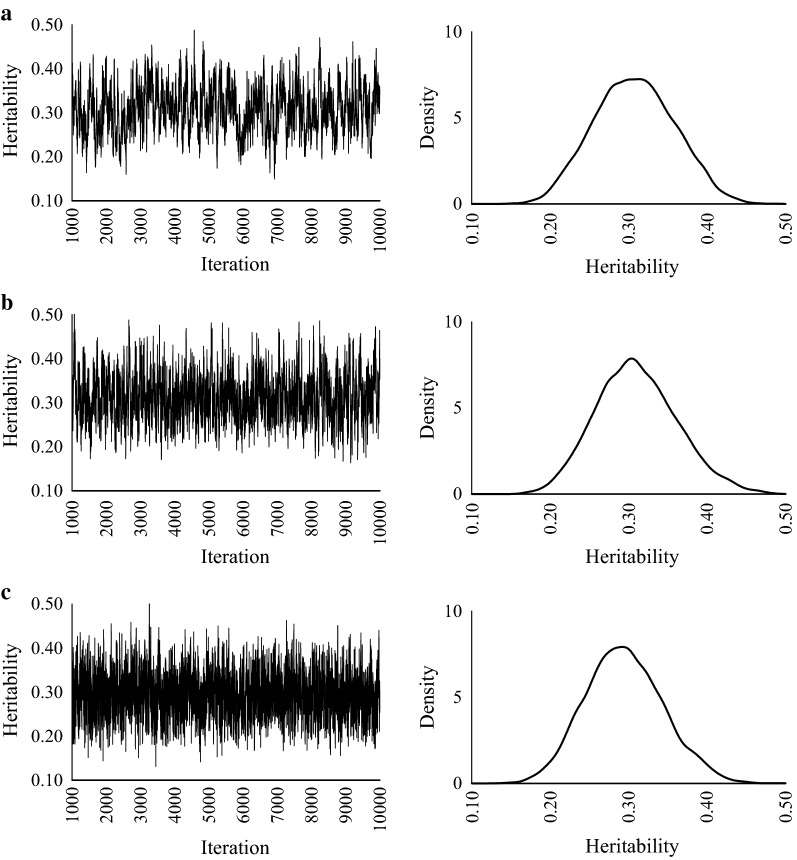
Trace plots and posterior density plots for heritability. **a** GS, **b** HMC, and **c** NUTS. True heritability = 0.3

**Fig. 3:**
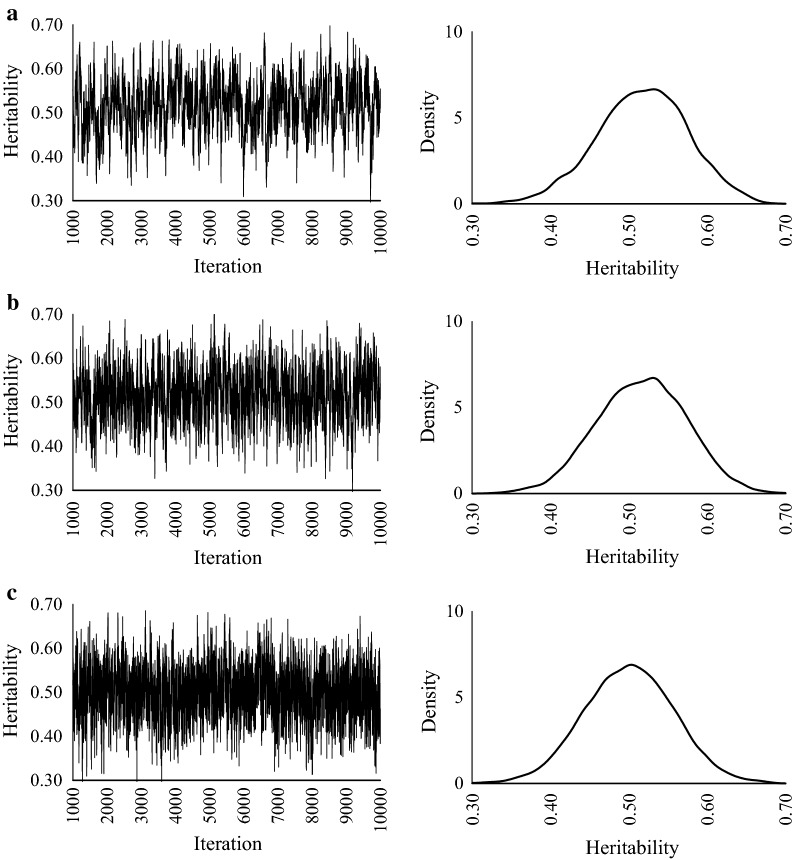
Trace plots and posterior density plots for heritability. **a** GS, **b** HMC, and **c** NUTS. True heritability = 0.5

The heritability estimates and predictive accuracies are in Table 2. Regardless of the values of true heritabilities, there are no differences in estimated heritabilities and accuracies among GS, HMC, and NUTS. The MCSE of NUTS was smallest for all scenarios. When the true heritability was 0.1, the marginal posterior distributions of GS and HMC were skewed compared with NUTS (Fig. 1). When true heritabilities were 0.3 and 0.5, the marginal posterior distributions for all methods were unimodal and bilaterally symmetrical (Figs. 2, 3).

**Table 2:** Estimates of heritabilities and breeding values in simulated data

True heritability	Method	Estimate			
		Heritability		Breeding value	
		Mean	MCSE	Correlation	Regression
0.1	GS	0.10	0.005	0.52	0.99
	HMC	0.11	0.004	0.52	0.99
	NUTS	0.10	0.001	0.53	1.10
0.3	GS	0.32	0.004	0.68	0.91
	HMC	0.31	0.002	0.68	0.93
	NUTS	0.30	0.001	0.68	0.96
0.5	GS	0.51	0.004	0.79	0.98
	HMC	0.52	0.003	0.79	0.97
	NUTS	0.51	0.002	0.79	0.99

*MCSE* Monte Carlo standard error, *GS* Gibbs sampling, *HMC* Hamiltonian Monte Carlo, *NUTS* No-U-Turn Sampler

Compared with the ESS estimates by GS, the ESS estimates by HMC and NUTS were 1.7 to 2.0 and 3.2 to 22.6 times larger, respectively (Table 3). The autocorrelation estimates by HMC and NUTS decreased more quickly than those by GS for all scenarios.

**Table 3:** Estimated effective sample size and autocorrelation in simulated data

True heritability	Method	ESS	Autocorrelation			
			Lag1	Lag5	Lag10	Lag50
0.1	GS	53	0.97	0.93	0.86	0.54
	HMC	91	0.96	0.88	0.79	0.36
	NUTS	1196	0.64	0.26	0.10	0.00
0.3	GS	204	0.93	0.78	0.62	0.08
	HMC	413	0.84	0.61	0.39	− 0.06
	NUTS	1192	0.64	0.28	0.11	0.00
0.5	GS	263	0.93	0.74	0.57	0.05
	HMC	462	0.84	0.57	0.37	− 0.03
	NUTS	833	0.72	0.38	0.18	0.05

*GS* Gibbs sampling, *HMC* Hamiltonian Monte Carlo, *NUTS* No-U-Turn Sampler

### Pig data

**Fig. 4:**
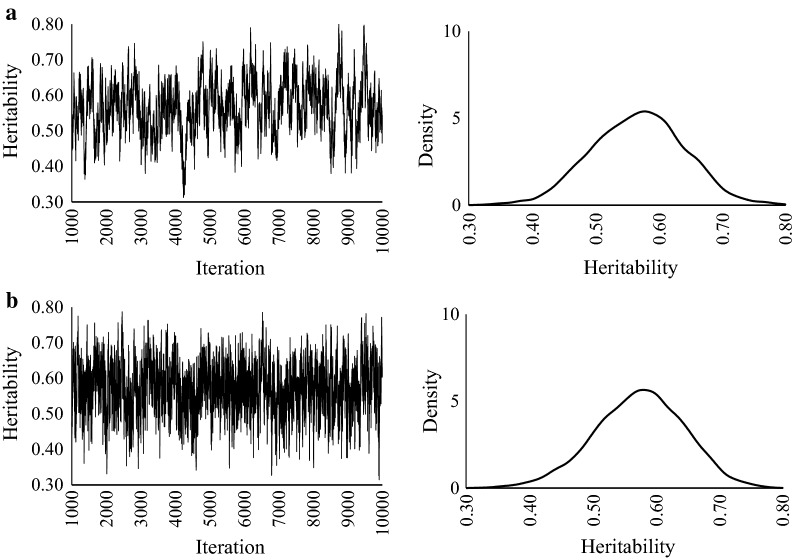
Trace plots and posterior density plots for heritability of loin eye area. **a** GS and **b** NUTS

**Fig. 5:**
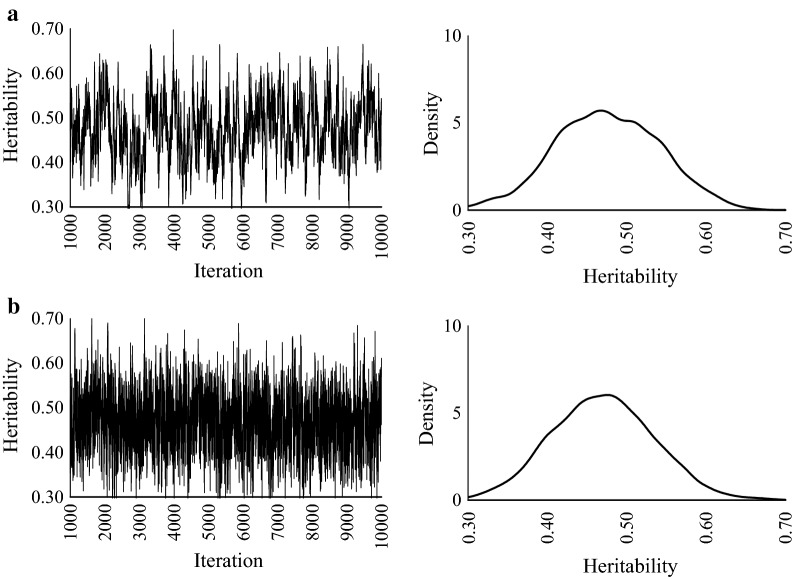
Trace plots and posterior density plots for heritability of backfat thickness. **a** GS and **b** NUTS

For LEA and BF, the heritability estimates and predictive accuracies of GS and NUTS were almost the same (Table 4). On the one hand, the MCSE of NUTS was smaller than that of GS for both LEA and BF. The marginal posterior distributions of these methods were unimodal and bilaterally symmetrical (Figs. 4, 5). On the other hand, HMC could not estimate heritability or breeding value because the parameters did not converge. Compared with the ESS estimates by GS, the ESS estimates by NUTS-Stan were 3.5 to 5.9 times larger (Table 5). The autocorrelation estimates of NUTS decreased more quickly than those of GS for both LEA and BF.

**Table 4:** Estimates of heritabilities and breeding values in Duroc pig data

Trait	Method	Estimate			
		Heritability		Breeding value	
		Mean	MCSE	Correlation	Regression
LEA	GS	0.56	0.006	0.82	1.60
	NUTS	0.57	0.003	0.84	1.59
BF	GS	0.48	0.006	0.65	1.37
	NUTS	0.47	0.002	0.66	1.47

*MCSE* Monte Carlo standard error, *BF* back fat thickness, *LEA* loin eye area, *GS* Gibbs sampling, *NUTS* No-U-Turn Sampler

**Table 5:** Estimated effective sample size and autocorrelation in Duroc pig data

Trait	Method	ESS	Autocorrelation			
			Lag1	Lag5	Lag10	Lag50
LEA	GS	132	0.96	0.85	0.75	0.30
	NUTS	457	0.82	0.57	0.35	0.06
BF	GS	151	0.95	0.83	0.70	0.21
	NUTS	886	0.68	0.36	0.17	0.04

*BF* back fat thickness, *LEA* loin eye area, *ESS* effective sample size, *GS* Gibbs sampling, *NUTS* No-U-Turn Sampler

## Discussion

### Performance of HMC and NUTS

This study examined the performance of HMC and NUTS, which can automatically optimize key parameters in HMC, for estimating heritabilities and breeding values as well as the quality of MCMC sampling in a pedigree-based univariate animal model. On the one hand, for both simulated and real pig data, NUTS performed better than GS. Compared with those by GS, the ESS and the declines of autocorrelation estimates by NUTS were larger and faster, respectively. In particular, when true heritability was low in simulated data, the skewness of the marginal posterior distributions of NUTS was smaller than that of GS. Thus, the results of the present study indicate that NUTS is an appropriate alternative sampling method for animal breeding. On the other hand, HMC could not estimate parameters for real data, whereas the performance of HMC was better than that of GS for simulated data. Therefore, it might be difficult to set appropriate hyperparameters for HMC according to trait and population structure.

### Computation time

R language is highly extensible and provides a myriad of statistical and graphical techniques. However, R language has poor computation time compared to Fortran, which is especially well suited to numeric computation and scientific computing. In the present study, we developed the programs for GS and HMC in R but did not examine computation time; instead, we focused on examining the performance of estimating genetic parameters and breeding values. In practice, the computation time of HMC and NUTS is greatly affected by leapfrog integration. In HMC, if $L$ is large, the computation load of leapfrog integration is large, although the distance of parameter transition is also large. In NUTS, candidate parameters must be calculated by leapfrog integration at all steps of the doubling procedure. Therefore, if the number of these steps increases, then the computation load increases exponentially. In the present study, the autocorrelations of HMC and NUTS decreased quickly compared to GS, which led to fast convergence. Thus, the computation time for HMC and NUTS can be shortened by reducing the number of MCMC iterations.

### Other sophisticated algorithms

Many algorithms that extend HMC have been published. Although NUTS is the most popular algorithm and is very effective for the sampling process, it can be slow if the evaluation of the gradient is computationally expensive, especially when large datasets are used. This problem might be solved by using stochastic gradient HMC [22]. Rather than directly computing the costly gradient, which requires examination of the entire dataset, stochastic gradient HMC uses a noisy estimate based on a small dataset, called minibatch, which is sampled uniformly at random from the entire dataset; as the size of minibatch increases, this approximation becomes more accurate. Empirically, in a variety of settings, simply considering a minibatch size on the order of hundreds of data points is sufficient to obtain an accurate estimate [23]. Minibatches of this size still represent a significant reduction in the computational cost of the gradient. Another algorithm that extends HMC is Riemannian manifold HMC [24]. Riemannian manifold HMC uses Riemann geometry to adapt the mass matrix, enabling the algorithm to use curvature information to perform more efficient sampling. The sampler will automatically adjust its movement through the probability space to better match the target distribution by using an appropriate metric for the manifold, thus providing highly efficient convergence and exploration of the target density. Hence, like NUTS, these extended HMC methods are also promising for the field of animal breeding.

## Conclusions

In our study, we compared the performance of GS, HMC, and NUTS for estimating genetic parameters and breeding values with both simulated and real pig data. The accuracies of NUTS were very similar to GS but NUTS had larger ESS estimates in the same iteration and declined more quickly for autocorrelations then GS. In addition, when true heritability was low in the simulated data, the skewness of the marginal posterior distributions of NUTS was smaller than that of GS. These results indicated that NUTS was an appropriate alternative sampling method for animal breeding. In particular, the performance for NUTS coded by using the Stan software was remarkably superior to other methods when true heritability was low in the simulated data. Furthermore, Stan’s simple programming language makes it quite practical for such applications. HMC could not estimate parameters for real data, whereas the performance of HMC was better than that of GS for simulated data, indicating that HMC requires appropriate parameter settings according to trait and population structure. Here, we applied HMC and NUTS to a univariate linear mixed model, thus future studies should investigate the possibility of applying HMC and NUTS to more complex models and big data.

## Supplementary information

**Additional file 1.** RStan code for the NUTS algorithm. RStan is the R interface to Stan. The user writes the analysis model in the test.stan file and runs Stan by using test.stan in R. The user needs to input the following parameters; J: number of fixed effect, K: number of all animals, N: number of observations, X: fixed effects design matrix, Z: random effects design matrix, Y: response variable, A: relationship matrix.

**Additional file 2.** Simulated data with a heritability of 0.1.

**Additional file 3.** Simulated data with a heritability of 0.3.

**Additional file 4.** Simulated data with a heritability of 0.5.

## Data Availability

Simulated data generated or analyzed during this study are included in Additional files 2, 3, and 4. The Duroc pig data are owned by the National Livestock Breeding Center in Japan and are not publicly available.
